# Robust detection and verification of linear relationships to generate metabolic networks using estimates of technical errors

**DOI:** 10.1186/1471-2105-8-162

**Published:** 2007-05-21

**Authors:** Frank Kose, Jan Budczies, Matthias Holschneider, Oliver Fiehn

**Affiliations:** 1 Universitaet Potsdam, D-14415 Potsdam, Germany; 2 Institute of Pathology, Charité University Hospital and provitro GmbH, D-10117 Berlin, Germany; 3 Universitaet Potsdam, Inst. f. Mathematik, D-14415 Potsdam, Germany; 4 University of California Davis, Genome Center, Davis CA 95616, USA

## Abstract

**Background:**

The size and magnitude of the metabolome, the ratio between individual metabolites and the response of metabolic networks is controlled by multiple cellular factors. A tight control over metabolite ratios will be reflected by a linear relationship of pairs of metabolite due to the flexibility of metabolic pathways. Hence, unbiased detection and validation of linear metabolic variance can be interpreted in terms of biological control. For robust analyses, criteria for rejecting or accepting linearities need to be developed despite technical measurement errors. The entirety of all pair wise linear metabolic relationships then yields insights into the network of cellular regulation.

**Results:**

The Bayesian law was applied for detecting linearities that are validated by explaining the residues by the degree of technical measurement errors. Test statistics were developed and the algorithm was tested on simulated data using 3–150 samples and 0–100% technical error. Under the null hypothesis of the existence of a linear relationship, type I errors remained below 5% for data sets consisting of more than four samples, whereas the type II error rate quickly raised with increasing technical errors. Conversely, a filter was developed to balance the error rates in the opposite direction. A minimum of 20 biological replicates is recommended if technical errors remain below 20% relative standard deviation and if thresholds for false error rates are acceptable at less than 5%. The algorithm was proven to be robust against outliers, unlike Pearson's correlations.

**Conclusion:**

The algorithm facilitates finding linear relationships in complex datasets, which is radically different from estimating linearity parameters from given linear relationships. Without filter, it provides high sensitivity and fair specificity. If the filter is activated, high specificity but only fair sensitivity is yielded. Total error rates are more favorable with deactivated filters, and hence, metabolomic networks should be generated without the filter. In addition, Bayesian likelihoods facilitate the detection of multiple linear dependencies between two variables. This property of the algorithm enables its use as a discovery tool and to generate novel hypotheses of the existence of otherwise hidden biological factors.

## Background

In recent years, time course analyses of metabolic perturbations have become more important to understand metabolic networks based on experimental data [1,2]. One way to analyze metabolic networks is by systematically investigating linear relationships between all analyzed metabolites (variables) followed by constructing networks from positively identified components, and eventually comparing network topologies [3] between different physiological or genetic conditions [4,5]. Simulations of metabolic reactions have shown that even stochastic influences on metabolism may result in linear metabolic co-regulation because initial metabolic perturbations can be propagated and enhanced through the cellular biochemical network [6]. Such linear co-regulation of pairs of metabolites may point to changes in biochemical control (chemical equilibrium, mass conservation, asymmetric control distribution) as well to transcriptional regulation [7]. Variance in metabolite levels can be caused by three different factors:

(I) concentrations alter and hence increase variance due to intentionally changing the experimental conditions, for example by altering environmental parameters like external nutrients or by using different genotypes [8],

(II) metabolite data will found to vary in a stochastic manner caused by the imprecision of the analytical method [9] used for acquiring metabolite data and

(III) interestingly, even under very controlled environmental conditions, a high degree of biological variation is found for metabolite levels due to stochastic biological events that trickle through the biochemical network and thus reflect the underlying control structure at this particular biological condition [6].

Therefore, if enough biological replicates are analyzed for a given organism at a given physiological situation, the metabolic phenotype can be investigated not only by its corresponding average metabolic values, but also by a snapshot of its corresponding metabolic network. However, biologists often do not know the inherent biological variability in advance and hence tend to use just a few independent biological replicates based on preliminary power analysis. Resulting data may be sufficient to estimate arithmetic means of metabolic levels but do not enable analyzing the linear control structure between different biological conditions. One of the challenges for calculating linearity networks is to compute the likelihood or significance of the presence of a truly linear relationship, with the aim of excluding both false negative and false positive detections of linearities.

Estimating optimal linearity parameters has been solved decades ago for cases, for which linear dependence of variables could be reasoned based on background knowledge. However, in metabolic data sets, the control structure of metabolites is unknown *a priori*. Therefore, two fundamental questions need to be answered:

(a) For which pairs of variables can a linear relationship be hypothesized?

(b) Are there sub sets of data that reflect differences in linear behavior of variables? For example, linearity may be given for only a group of data but absent in another group, or the linearity parameters between these groups may be different.

An unbiased analysis of linear relationships between pairs of variables needs to test whether there is one or more valid linear hypotheses that could explain data in complex data sets. This procedure defines a novel approach for testing biological data: instead testing pre-defined hypotheses [10], the likelihood of hypotheses is calculated that may be used to explain complex process. This hypothesis-building is fundamentally different from estimating the best parameters of an assumed linear relationship by regression equations [11-13] for which various software packages exist, and solutions for estimating parameters for multiple linear relationships[14]. However, regressions do not test the probability of the presence of linear relationships, especially in high-dimensional data sets. Instead, regressions are founded on the presence of linearity that is justified by background knowledge. In biochemistry, the existence of linear relationships cannot generally be assumed trivial but must receive thorough statistical evaluations. In addition, regressions usually do not account for technical errors [15] that are critical in practice. All measurements comprise technical errors which are due to inadequacy of the total chemical-analytical method, specifically the extraction, sample preparation and the instrumental data acquisition. Hence, the degree of technical errors will vary between the chemical nature of the metabolites, their absolute concentrations and influences of different sample matrices. Furthermore, outliers and missing data further obscure detection of linear hypotheses. For regressions, on the other hand, the impact of outliers has been studied extensively, and multiple measures to assess and weigh the influence of outliers have been developed. Assumptions on the degree of technical errors may further refine weighting factors in regression analysis, and such factors can be optimized for example using the EM-algorithm. Nevertheless, regression is not an explorative tool for data analysis. Additionally, metabolomic data do not distinguish between dependent and independent variables. All variables are subject to varying degree of noise (analytical-chemical measurement errors, i.e. technical errors). No controlled observations of supposedly independent variables can be acquired [16,17]. Consequently, the control structure of metabolic linearity networks can only be assessed with a tool that solves the following tasks:

(1) Linear relationships must be detected in an unbiased and observer-independent manner.

(2) Sub sets of data need to be grouped according to presence of (multiple) linear relationships.

(3) Criteria have to be applied that verify linear hypotheses based on test statistics.

(4) Technical errors: varying degree of analytical-chemical measurement errors and missing data have to be accounted for.

Especially, the potential presence of multiple linear relationships and independence of both variables poses problems for simple regression analyses. As a substitute for regression, the degree of correlation has been used for detecting linear relationships despite the fact that correlation only relates the covariance to the total variance, but does not verify genuine linearities. Moreover, Pearsons' correlation coefficients lack robustness against outliers, especially for multivariate datasets, and a number of different approaches have been suggested to link estimates to better test statistics [18]. In practice, however, empirical or heuristic thresholds are taken to distinguish strong or weak correlations, but no mathematical basis exists on which such thresholds can safely be founded. In some cases, Student's statistics *p*-values have been taken in an effort to validate Pearson's correlations [19]. Unfortunately, such *p*-values only describe the significance of the non-randomness of data pairs but do not test hypotheses if data pairs can be described by a (single or multiple) linear functions. Consequently, correlation networks based on Pearsons correlations may be strongly distorted [20].

A further approach has been taken using partial correlations that deconvolute contributions by additional parameters in order to reduce the list of correlations to basic dependencies [21] which may present a link from correlation to causality [22,23]. This method is valuable to investigate the control structure within a given correlation network but it does not remove the principle robustness problem of correlation estimates. Simple correlations coefficients always decline with increasing variance that is introduced by method errors during data acquisition. In contrary, partial correlation coefficients may be increasing, decreasing or even change the algebraic signs with increasing method errors [20]. In order to remedy this situation, scientists tend to select high Pearson's correlation thresholds [24] which imply that the variance caused by method errors is small in relation to the biological variance. The latter assumption is often true when comparing widely different metabolic phenotypes such as certain mutant genotypes, or severe stress conditions such as acute (metabolic) diseases in comparison to healthy states. However, metabolic theory predicts that even incremental changes in enzymatic properties can have large effects on metabolic control, especially when multiple enzymes are affected [25]. Such changes might be too subtle to cause large differences in average concentrations but would still effect the pathway control structure and hence, linearities in pairs of metabolite data. Consequently, the metabolic control structure can only be assessed with a robust tool for linearity detection.

We here present a different approach. Using the Bayesian law [26], a likelihood formula is derived that is based on information about the measurement error using a specific technical method. This formula is then transformed in way that allows searching for local maxima of linear parameters within the total hypothesis space. Such likelihood maxima are subsequently assessed for residuals of the corresponding linearity parameters using simulated test statistics. We demonstrate the power of this approach using a synthetic data set with a given set of true linear relationships which are subsequently subjected to both increasing technical errors and increasing number of samples.

## Results and Discussion

### (1) A model for the technical error in metabolomic data

Let {*x*_*ij *_} denote the entirety of *n *metabolite measurements in a collection of *m *samples. The measurements can be arranged in a matrix *x*_*ij *_with rows i = 1, ..., *n *that refer to the metabolites and columns j = 1, ..., *m *that refer to the samples. Each measurement results from the sum of the true metabolite content x′ij
 MathType@MTEF@5@5@+=feaafiart1ev1aaatCvAUfKttLearuWrP9MDH5MBPbIqV92AaeXatLxBI9gBaebbnrfifHhDYfgasaacH8akY=wiFfYdH8Gipec8Eeeu0xXdbba9frFj0=OqFfea0dXdd9vqai=hGuQ8kuc9pgc9s8qqaq=dirpe0xb9q8qiLsFr0=vr0=vr0dc8meaabaqaciaacaGaaeqabaqabeGadaaakeaacuWG4baEgaqbamaaBaaaleaacqWGPbqAcqWGQbGAaeqaaaaa@3115@ and a technical error,

xij=x′ij+eij.
 MathType@MTEF@5@5@+=feaafiart1ev1aaatCvAUfKttLearuWrP9MDH5MBPbIqV92AaeXatLxBI9gBaebbnrfifHhDYfgasaacH8akY=wiFfYdH8Gipec8Eeeu0xXdbba9frFj0=OqFfea0dXdd9vqai=hGuQ8kuc9pgc9s8qqaq=dirpe0xb9q8qiLsFr0=vr0=vr0dc8meaabaqaciaacaGaaeqabaqabeGadaaakeaacqWG4baEdaWgaaWcbaGaemyAaKMaemOAaOgabeaakiabg2da9iqbdIha4zaafaWaaSbaaSqaaiabdMgaPjabdQgaQbqabaGccqGHRaWkcqWGLbqzdaWgaaWcbaGaemyAaKMaemOAaOgabeaakiabc6caUaaa@3C93@

The technical errors *e*_*ij *_include the chemical-analytical error, but can also include a contribution from different storage manners or times of the biomaterial after its extraction. The technical variance of the j*th *measurement can be derived by a probability density function *ρ*_*j *_that reflects knowledge about sample storage and data acquisition. For missing data, it is only known that these can be expected in a defined range but with uniform probability distribution. For non-missing data, the technical error is modeled by a multivariate normal distribution that is centered around zero. More precisely, the probability density for the technical error *e*_*j *_= (*e*_1 *j *_,..., *e*_*nj*_)^*t *^of the *j*th metabolic profile is given by

ρj(ej)=Nexp⁡(−12ejt∑jej−1).

In principle, the variance matrices Σ_*j *_can be estimated from the covariance matrix of replicated measurements of the same biomaterial. In practice, correlations between the technical errors of different metabolites are often disregarded, leading to a model with diagonal variance matrices Σj=diag(σ1j2,...,σnj2)
 MathType@MTEF@5@5@+=feaafiart1ev1aaatCvAUfKttLearuWrP9MDH5MBPbIqV92AaeXatLxBI9gBaebbnrfifHhDYfgasaacH8akY=wiFfYdH8Gipec8Eeeu0xXdbba9frFj0=OqFfea0dXdd9vqai=hGuQ8kuc9pgc9s8qqaq=dirpe0xb9q8qiLsFr0=vr0=vr0dc8meaabaqaciaacaGaaeqabaqabeGadaaakeaacqqHJoWudaWgaaWcbaGaemOAaOgabeaakiabg2da9iabbsgaKjabbMgaPjabbggaHjabbEgaNnaabmaabaacciGae83Wdm3aa0baaSqaaiabigdaXiabdQgaQbqaaiabikdaYaaakiabcYcaSiabc6caUiabc6caUiabc6caUiabcYcaSiab=n8aZnaaDaaaleaacqWGUbGBcqWGQbGAaeaacqaIYaGmaaaakiaawIcacaGLPaaaaaa@46ED@. Further one often works with fixed absolute or fixed relative errors,

σij(abs.)=ai or σij(rel.)=rixij,
 MathType@MTEF@5@5@+=feaafiart1ev1aaatCvAUfKttLearuWrP9MDH5MBPbIqV92AaeXatLxBI9gBaebbnrfifHhDYfgasaacH8akY=wiFfYdH8Gipec8Eeeu0xXdbba9frFj0=OqFfea0dXdd9vqai=hGuQ8kuc9pgc9s8qqaq=dirpe0xb9q8qiLsFr0=vr0=vr0dc8meaabaqaciaacaGaaeqabaqabeGadaaakeaaiiGacqWFdpWCdaqhaaWcbaGaemyAaKMaemOAaOgabaGaeiikaGIaeeyyaeMaeeOyaiMaee4CamNaeiOla4IaeiykaKcaaOGaeyypa0Jaemyyae2aaSbaaSqaaiabdMgaPbqabaGccqqGGaaicqqGVbWBcqqGYbGCcqqGGaaicqWFdpWCdaqhaaWcbaGaemyAaKMaemOAaOgabaGaeiikaGIaeeOCaiNaeeyzauMaeeiBaWMaeiOla4IaeiykaKcaaOGaeyypa0JaemOCai3aaSbaaSqaaiabdMgaPbqabaGccqWG4baEdaWgaaWcbaGaemyAaKMaemOAaOgabeaakiabcYcaSaaa@54E5@

respectively.

### (2) Maximum Likelihood (ML) function for a general linear problem

Using the Bayesian law, the likelihood for parameters of a given linear hypothesis can be calculated. The Bayesian law allows to interconvert the conditional probabilities of cause (linear relationship) and effect (measured data value) [27,28],

p(A|B)=p(B|A)p(A)p(B)
 MathType@MTEF@5@5@+=feaafiart1ev1aaatCvAUfKttLearuWrP9MDH5MBPbIqV92AaeXatLxBI9gBaebbnrfifHhDYfgasaacH8akY=wiFfYdH8Gipec8Eeeu0xXdbba9frFj0=OqFfea0dXdd9vqai=hGuQ8kuc9pgc9s8qqaq=dirpe0xb9q8qiLsFr0=vr0=vr0dc8meaabaqaciaacaGaaeqabaqabeGadaaakeaacqWGWbaCcqGGOaakcqWGbbqqcqGG8baFcqWGcbGqcqGGPaqkcqGH9aqpdaWcaaqaaiabdchaWjabcIcaOiabdkeacjabcYha8jabdgeabjabcMcaPiabdchaWjabcIcaOiabdgeabjabcMcaPaqaaiabdchaWjabcIcaOiabdkeacjabcMcaPaaaaaa@4376@

The general form of a linear relationship in the metabolomics data is

α1x′ij+...+αnx′nj=βfor all samplesj=1,...,m.
 MathType@MTEF@5@5@+=feaafiart1ev1aaatCvAUfKttLearuWrP9MDH5MBPbIqV92AaeXatLxBI9gBaebbnrfifHhDYfgasaacH8akY=wiFfYdH8Gipec8Eeeu0xXdbba9frFj0=OqFfea0dXdd9vqai=hGuQ8kuc9pgc9s8qqaq=dirpe0xb9q8qiLsFr0=vr0=vr0dc8meaabaqaciaacaGaaeqabaqabeGadaaakeaafaqabeqadaaabaacciGae8xSde2aaSbaaSqaaiabigdaXaqabaGccuWG4baEgaqbamaaBaaaleaacqWGPbqAcqWGQbGAaeqaaOGaey4kaSIaeiOla4IaeiOla4IaeiOla4Iaey4kaSIae8xSde2aaSbaaSqaaiabd6gaUbqabaGccuWG4baEgaqbamaaBaaaleaacqWGUbGBcqWGQbGAaeqaaOGaeyypa0Jae8NSdigabaGaeeOzayMaee4Ba8MaeeOCaiNaeeiiaaIaeeyyaeMaeeiBaWMaeeiBaWMaeeiiaaIaee4CamNaeeyyaeMaeeyBa0MaeeiCaaNaeeiBaWMaeeyzauMaee4CamhabaGaemOAaOMaeyypa0JaeGymaeJaeiilaWIaeiOla4IaeiOla4IaeiOla4IaeiilaWIaemyBa0MaeiOla4caaaaa@6017@

In what follows we collect the coefficients of the above equation in a vector *α *= (*α*_1_,..., *α*_*n*_)^*t *^and express the linear relationship as *α*^*t *^*x*_•*j *_= *β*. By *x*_•*j *_we denote the metabolic profile of the *j*th sample. The entirety of the parameters *α *and *β *results in *A*. The probability *p *(*A*) is the *a priori *probability of the parameters *α *and *β *before the measurement has been performed. Because no preference can be given, the *a priori *probability is constant for all *A*. The same is true for *p *(*B*) with *B *representing the measured metabolite concentrations. Therefore, *p *(*A *| *B*) is the likelihood for parameter *A *if the pair of variables *B *is given. We have used an unbiased approach here assuming random and unrelated technical errors, and we cannot know beforehand if a certain metabolite will be detectable or not, and how large the concentration of such metabolite could be. These assumptions result in a constant probability for p(A) and p(B), because otherwise certain values for A and B would be more likely than others. From the Bayesian law it can be concluded

*p *(*A *| *B*) = *c *· *p *(*B *| *A*).

The constant value *c *can be neglected because the objective is to compare different linear hypotheses. Consequently, a hypothetical metabolic profile has the same probability at a given linear hypothesis as a hypothetical linear hypothesis at a given metabolic profile. The expression *p *(*B*|*A*) is therefore the probability that the measured metabolic profile *B *is determined at a given set of parameters *A*, which can be calculated using the function which describes the probability distribution of the technical error. We are now in position to state the following general theorem:

*Let x *= (*x*_1_, ..., *x*_*n*_)^*t *^*include the measurements of n metabolites in a biological sample with technical errors that follow a Gaussian distribution with covariance matrix Σ. Let a (n-N)-dimensional surface in the n-dimensional metabolite space be defined by the equations*

*α*_*k*1 _*x*_1 _+ ... + *α*_*kn *_*x*_*n *_= *β*_*k*_   *for k *= 1, ..., *N*.

*The coefficients of these equations comprise a matrix α = (α_*ki*_) and a vector β = (β_1_, ..., β_*N*_)^*t*^. The matrix elements can also be arranged in vectors α*_*k *_:(*α*_*k*1_, ..., *α*_*kn*_)^*t*^. *It is assumed the hyperplanes defined by (8) are orthogonal in pairs with respect to the covariance matrix, i.e*.

*α*_*k *_^*t*^Σ *α*_*l *_= 0   *for k*, *l *= 1, ..., *N and k *≠ *l*.

Then, the likelihood for the metabolite concentrations to lie on the (n-k)-dimensional surface is given by

p(x|α,β)=exp⁡(−12∑k=1N(αktx−βk)2αktΣαk).
 MathType@MTEF@5@5@+=feaafiart1ev1aaatCvAUfKttLearuWrP9MDH5MBPbIqV92AaeXatLxBI9gBaebbnrfifHhDYfgasaacH8akY=wiFfYdH8Gipec8Eeeu0xXdbba9frFj0=OqFfea0dXdd9vqai=hGuQ8kuc9pgc9s8qqaq=dirpe0xb9q8qiLsFr0=vr0=vr0dc8meaabaqaciaacaGaaeqabaqabeGadaaakeaacqWGWbaCcqGGOaakcqWG4baEcqGG8baFiiGacqWFXoqycqGGSaalcqWFYoGycqGGPaqkcqGH9aqpcyGGLbqzcqGG4baEcqGGWbaCdaqadaqaaiabgkHiTmaalaaabaGaeGymaedabaGaeGOmaidaamaaqahabaWaaSaaaeaadaqadaqaaiab=f7aHnaaDaaaleaacqWGRbWAaeaacqWG0baDaaGccqWG4baEcqGHsislcqWFYoGydaWgaaWcbaGaem4AaSgabeaaaOGaayjkaiaawMcaamaaCaaaleqabaGaeGOmaidaaaGcbaGae8xSde2aa0baaSqaaiabdUgaRbqaaiabdsha0baakiabfo6atjab=f7aHnaaBaaaleaacqWGRbWAaeqaaaaaaeaacqWGRbWAcqGH9aqpcqaIXaqmaeaacqWGobGta0GaeyyeIuoaaOGaayjkaiaawMcaaiabc6caUaaa@5E67@

The theorem is proven in Additional File 1. However, the result for the likelihood has a simple interpretation: it is proportional to the density of the normal distribution taken at the distance of the measurement from the surface. This distance has to be calculated by taking the covariance matrix of the technical errors as metric of the metabolite space. Next, let us illustrate the theorem by a special case that is especially interesting for applications: Consider two metabolite concentrations that are measured with technical standard deviations *σ*_1_, *σ*_2 _and technical covariance *σ*_12_. Then, the likelihood for the metabolite concentration to lie on the straight line *α*_1 _*x*_1 _+ *α*_2 _*x*_2 _= *β *is given by

p(x1,x2|α1,α2,β)=exp⁡(−12(α1x1+α2x2−β)2σ12α12+2σ12α1α2+σ22α22).
 MathType@MTEF@5@5@+=feaafiart1ev1aaatCvAUfKttLearuWrP9MDH5MBPbIqV92AaeXatLxBI9gBaebbnrfifHhDYfgasaacH8akY=wiFfYdH8Gipec8Eeeu0xXdbba9frFj0=OqFfea0dXdd9vqai=hGuQ8kuc9pgc9s8qqaq=dirpe0xb9q8qiLsFr0=vr0=vr0dc8meaabaqaciaacaGaaeqabaqabeGadaaakeaacqWGWbaCcqGGOaakcqWG4baEdaWgaaWcbaGaeGymaedabeaakiabcYcaSiabdIha4naaBaaaleaacqaIYaGmaeqaaOGaeiiFaWhcciGae8xSde2aaSbaaSqaaiabigdaXaqabaGccqGGSaalcqWFXoqydaWgaaWcbaGaeGOmaidabeaakiabcYcaSiab=j7aIjabcMcaPiabg2da9iGbcwgaLjabcIha4jabcchaWnaabmaabaGaeyOeI0YaaSaaaeaacqaIXaqmaeaacqaIYaGmaaWaaSaaaeaadaqadaqaaiab=f7aHnaaBaaaleaacqaIXaqmaeqaaOGaemiEaG3aaSbaaSqaaiabigdaXaqabaGccqGHRaWkcqWFXoqydaWgaaWcbaGaeGOmaidabeaakiabdIha4naaBaaaleaacqaIYaGmaeqaaOGaeyOeI0Iae8NSdigacaGLOaGaayzkaaWaaWbaaSqabeaacqaIYaGmaaaakeaacqWFdpWCdaqhaaWcbaGaeGymaedabaGaeGOmaidaaOGae8xSde2aa0baaSqaaiabigdaXaqaaiabikdaYaaakiabgUcaRiabikdaYiab=n8aZnaaBaaaleaacqaIXaqmcqaIYaGmaeqaaOGae8xSde2aaSbaaSqaaiabigdaXaqabaGccqWFXoqydaWgaaWcbaGaeGOmaidabeaakiabgUcaRiab=n8aZnaaDaaaleaacqaIYaGmaeaacqaIYaGmaaGccqWFXoqydaqhaaWcbaGaeGOmaidabaGaeGOmaidaaaaaaOGaayjkaiaawMcaaiabc6caUaaa@76EC@

Returning to the general line of the text and the Bayesian reasoning we obtain for the likelihood for a linear relationship described by *A *= {*α*_*ki*_, *β*_*k*_} after measurement of the metabolomic data *B *= {*x*_*ij*_} the result

p(A|B)=∏j=1mlj(α,β|x·j)
 MathType@MTEF@5@5@+=feaafiart1ev1aaatCvAUfKttLearuWrP9MDH5MBPbIqV92AaeXatLxBI9gBaebbnrfifHhDYfgasaacH8akY=wiFfYdH8Gipec8Eeeu0xXdbba9frFj0=OqFfea0dXdd9vqai=hGuQ8kuc9pgc9s8qqaq=dirpe0xb9q8qiLsFr0=vr0=vr0dc8meaabaqaciaacaGaaeqabaqabeGadaaakeaacqWGWbaCcqGGOaakcqWGbbqqcqGG8baFcqWGcbGqcqGGPaqkcqGH9aqpdaqeWbqaaiabdYgaSnaaBaaaleaacqWGQbGAaeqaaOGaeiikaGccciGae8xSdeMaeiilaWIae8NSdiMaeiiFaWNaemiEaG3aaSbaaSqaaiabl+y6NjabdQgaQbqabaGccqGGPaqkaSqaaiabdQgaQjabg2da9iabigdaXaqaaiabd2gaTbqdcqGHpis1aaaa@4B10@

with

lj(α,β|x·j)=p(x·j|α,β)=exp⁡(−12∑k=1N(αktx·j−βk)2αktΣjαk)
 MathType@MTEF@5@5@+=feaafiart1ev1aaatCvAUfKttLearuWrP9MDH5MBPbIqV92AaeXatLxBI9gBaebbnrfifHhDYfgasaacH8akY=wiFfYdH8Gipec8Eeeu0xXdbba9frFj0=OqFfea0dXdd9vqai=hGuQ8kuc9pgc9s8qqaq=dirpe0xb9q8qiLsFr0=vr0=vr0dc8meaabaqaciaacaGaaeqabaqabeGadaaakeaacqWGSbaBdaWgaaWcbaGaemOAaOgabeaakiabcIcaOGGaciab=f7aHjabcYcaSiab=j7aIjabcYha8jabdIha4naaBaaaleaacqWIpM+zcqWGQbGAaeqaaOGaeiykaKIaeyypa0JaemiCaaNaeiikaGIaemiEaG3aaSbaaSqaaiabl+y6NjabdQgaQbqabaGccqGG8baFcqWFXoqycqGGSaalcqWFYoGycqGGPaqkcqGH9aqpcyGGLbqzcqGG4baEcqGGWbaCdaqadaqaaiabgkHiTmaalaaabaGaeGymaedabaGaeGOmaidaamaaqahabaWaaSaaaeaadaqadaqaaiab=f7aHnaaDaaaleaacqWGRbWAaeaacqWG0baDaaGccqWG4baEdaWgaaWcbaGaeS4JPFMaemOAaOgabeaakiabgkHiTiab=j7aInaaBaaaleaacqWGRbWAaeqaaaGccaGLOaGaayzkaaWaaWbaaSqabeaacqaIYaGmaaaakeaacqWFXoqydaqhaaWcbaGaem4AaSgabaGaemiDaqhaaOGaeu4Odm1aaSbaaSqaaiabdQgaQbqabaGccqWFXoqydaWgaaWcbaGaem4AaSgabeaaaaaabaGaem4AaSMaeyypa0JaeGymaedabaGaemOta4eaniabggHiLdaakiaawIcacaGLPaaaaaa@77DA@

The corresponding likelihood function is a sum of contributions from each of the biological samples,

L(A|B)=ln⁡p(A|B)=∑j=1mln⁡lj(α,β|x·j)
 MathType@MTEF@5@5@+=feaafiart1ev1aaatCvAUfKttLearuWrP9MDH5MBPbIqV92AaeXatLxBI9gBaebbnrfifHhDYfgasaacH8akY=wiFfYdH8Gipec8Eeeu0xXdbba9frFj0=OqFfea0dXdd9vqai=hGuQ8kuc9pgc9s8qqaq=dirpe0xb9q8qiLsFr0=vr0=vr0dc8meaabaqaciaacaGaaeqabaqabeGadaaakeaacqWGmbatcqGGOaakcqWGbbqqcqGG8baFcqWGcbGqcqGGPaqkcqGH9aqpcyGGSbaBcqGGUbGBcqWGWbaCcqGGOaakcqWGbbqqcqGG8baFcqWGcbGqcqGGPaqkcqGH9aqpdaaeWbqaaiGbcYgaSjabc6gaUjabdYgaSnaaBaaaleaacqWGQbGAaeqaaOGaeiikaGccciGae8xSdeMaeiilaWIae8NSdiMaeiiFaWNaemiEaG3aaSbaaSqaaiabl+y6NjabdQgaQbqabaGccqGGPaqkaSqaaiabdQgaQjabg2da9iabigdaXaqaaiabd2gaTbqdcqGHris5aaaa@581E@

Maximizing *p *(*A*|*B*) or *L *(*A*|*B*) gives the maximum likelihood. The resulting estimator for the considered linear relationship is called the simple ML-estimator. The likelihood takes values between 0 and 1. Likelihoods are different to probabilities [29] with respect to *p *(*B*|*A*) which is maximized in order to find the most likely parameters for a given hypothesis, here: a linear hypothesis.

### (3) An adapted Maximum Likelihood estimator for robust verification of linear hypotheses

The product *p *(*B*|*A*) can never become larger than one of its factors, and it comprises exactly one global maximum. Consequently, just a single outlier may decrease *p *(*B*|*A*) significantly. Unfortunately, outlier data are frequently found in biological data sets due to both the multitude of factors in biological cells and the complexity of data acquisition methods that may result in false positive data points. Furthermore, it is still unclear, how many linear relationships exist for a given pair of variables. Both questions are reflected by introducing a decoupling term, the constant c, to the likelihood function,

Lc(A|B)=ln⁡pc(A|B)=∑j=1mln⁡(lj(α,β|x·j)+c)
 MathType@MTEF@5@5@+=feaafiart1ev1aaatCvAUfKttLearuWrP9MDH5MBPbIqV92AaeXatLxBI9gBaebbnrfifHhDYfgasaacH8akY=wiFfYdH8Gipec8Eeeu0xXdbba9frFj0=OqFfea0dXdd9vqai=hGuQ8kuc9pgc9s8qqaq=dirpe0xb9q8qiLsFr0=vr0=vr0dc8meaabaqaciaacaGaaeqabaqabeGadaaakeaacqWGmbatdaWgaaWcbaGaem4yamgabeaakiabcIcaOiabdgeabjabcYha8jabdkeacjabcMcaPiabg2da9iGbcYgaSjabc6gaUjabdchaWnaaBaaaleaacqWGJbWyaeqaaOGaeiikaGIaemyqaeKaeiiFaWNaemOqaiKaeiykaKIaeyypa0ZaaabCaeaacyGGSbaBcqGGUbGBcqGGOaakcqWGSbaBdaWgaaWcbaGaemOAaOgabeaakiabcIcaOGGaciab=f7aHjabcYcaSiab=j7aIjabcYha8jabdIha4naaBaaaleaacqWIpM+zcqWGQbGAaeqaaOGaeiykaKIaey4kaSIaem4yamMaeiykaKcaleaacqWGQbGAcqGH9aqpcqaIXaqmaeaacqWGTbqBa0GaeyyeIuoaaaa@5F0B@

Additional File 2 gives the impact of the magnitude of the constant *c *on the total likelihood. It is demonstrated in an empirical way that the total likelihood is not decreased by any *c *≥ 1. In what follows we fix the constant at the value *c *= 1 und consider an adapted ML-estimator that is constructed by maximization of *L*_1_(*A *| *B*).

**Figure 1:**
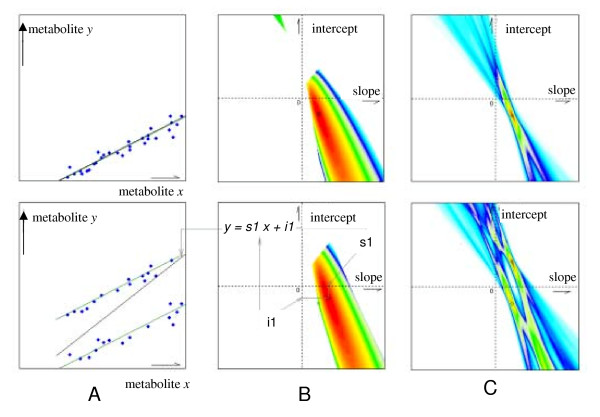
Comparison between simple and adapted maximum likelihood estimation. *Graph A*. The upper panel represents a set of 30 covariate pairs (‚samples‘) which can be described by a linear function. Deviation from this function is due to a simulated technical error. The lower panel comprises 30 samples for which half of the data were shifted for a constant value. *Graph B *Likelihood distribution for the hypotheses space using the simple maximum likelihood estimator using data from upper and lower panel from graph A. For any given linearity parameter (slope and intercept), the estimated likelihood is increasing from white to cyan, blue, gree, yellow, orange and red. Upper panel: For a single linearity, the global maximum (black circle) matches with the linearity parameters of the simulated function (green circle). Lower panel: The simple maximum likelihood estimator fails to detect and represent the presence of two linear functions. The global maximum is calculated for a single linearity which is depicted in graph A, lower panel. *Graph C*: Likelihood distribution for the hypotheses space using the simple maximum likelihood estimator using the same data set as in graphs A and B. A single linearity is correctly identified (upper panel). Importantly, data sets comprising more than one linear function are also correctly matched reporting both slope and intercept parameters.

The adapted likelihood function is a sum, to which every data point adds a contribution between zero and a maximum value of ln 2 if it coincides with the linear hypothesis that is under investigation. This step alters the impact of the Bayesian law. It results in assessing each individual variable pair by a likelihood of contribution to a (linear) hypothesis, and not by assessing the entirety of all variable pairs. Consequently, the contribution of outliers is evanescent as demonstrated in figure 1 and is limited to a reduction of *L *of ln 2 in the worst case. Figure 1 shows two plots, each representing 30 data pairs. In the upper panels, the 30 data pairs shall follow a hypothetical linear function with an additional modeled analytical measurement error. The lower panels represent a data set in which 15 of the 30 data points are transposed by a constant value, so that two linear functionalities exist. For each of the two examples, likelihood distributions are given across a part of the hypothesis space for the simple ML estimator (mid panels B) and the adapted ML estimator (right hand panels C). For the case of a dataset that comprised two likely linear functions, the simple ML estimator only recognizes a shift in the local maximum but fails to detect two local maxima according to the two linearities. In contrary, the adapted ML estimator correctly identifies both local maxima and is thus able to detect the most likely parameters for both linear functions. In fact, the local likelihood maximum of the original single linear function does not shift for the adapted ML estimator when 15 data points are shifted by a constant factor but it just leads to a decrease of the maximal possible likelihood. If all measured data are assigned to different linear hypotheses according to their corresponding likelihoods, the criterion (2) as given above in the section 'background' is fulfilled: Sub sets of data are now grouped according to presence of (multiple) linear relationships.

Additional considerations are outlined for the case of missing data (NANs, not-a-number) which are often found in metabolomic data sets. In such cases, the probability function *p *(*B*|*A*) needs to be adapted. This function then represents the information about the missing value: for example, data could get lost due to measurement instrument malfunctions or the variable (i.e. a metabolite level) might be below the limit of detection in a given biological situation. In both cases, the probability density function is uniform, i.e. the probability is constant in a certain range. For the case of 'below detection limit', the probability density is limited, for the case of 'instrument malfunction' the probability density is zero at all levels. However, for both cases, the undefined integral is one (we must assume a false negative metabolite detection). If we knew the true cause of the missing value (i.e. either false negative or true negative), the correct probability density function could be modeled. For now, however, we need to assume an infinite technical error which demands to add the maximal likelihood of ln2 to these data points. Accordingly, missing data do not have a diminishing impact on *L*. An extreme case of data set that exclusively comprises missing data would result in a maximal likelihood for arbitrary linear hypotheses. This interpretation is correct because all hypotheses would be equally probable and could not be denied, which, however, results to an interpretive power of zero. Consequently, for real cases a maximal number of missing values needs to be defined in order to deny any linear hypothesis that might be due to missing explanatory power. The upper limit of the number of such missing data has to be set by the user who may call in further biological or analytical background information for individual metabolite pairs.

Concluding, the following properties are observed for the adapted ML-estimator:

(i) The adapted ML-estimator considers technical errors.

(ii) The adapted ML-estimator detects linear patterns and groups sub sets of data accordingly.

(iii) The adapted ML-estimator is robust against outliers.

(iv) The adapted ML-estimator relies on background information on missing values and therefore does not distort interpretations.

Therefore, the adapted ML-estimator realizes a solution to several of the challenges of unbiased and robust detection of multiple linear hypotheses in complex data sets.

### (4) Algorithm for the detection of linear relationships

In order to assign measured data to a hypothetical linear relationship without contradictions, corresponding residues have to be analyzed. One condition is that these residues are randomly distributed; otherwise, additional systematic errors would have to be assumed. Secondly, the residues have to be explainable by the technical errors in a statistical manner. The adapted ML-estimator already realizes a measure for agreement between (linear) model and data under consideration of the corresponding technical errors. Thresholds can now be determined for rejecting specific linear hypotheses using the distribution of *L*, resulting in false discovery rates for which limits can be set. The core of the algorithm determines the local maximum of a likelihood distribution which is subsequently compared to limits of a test statistics. It can be assumed that this maximum will be the global maximum since all measured data will be explained by the tested linear relationship. However, outliers will reduce the likelihood drastically. Consequently, data are only considered if residues are small to the hypothetical linearity. The 2 *σ *interval was chosen to exclude outliers at 95% confidence. The data inside the 2 *σ *confidence interval is denoted by *B*_return_. The likelihood is subsequently normalized to the number *m*_return _of this data. The parameter m_return _comprises the number of samples that were returned to belong to a linear function despite deviation that is due to the contribution of unrelated variance. Each data point contributes a value of ln 2 to the likelihood function, resulting in the normalized likelihood

Lreturn=L1(Amax⁡|Breturn)mreturn×ln⁡2
 MathType@MTEF@5@5@+=feaafiart1ev1aaatCvAUfKttLearuWrP9MDH5MBPbIqV92AaeXatLxBI9gBaebbnrfifHhDYfgasaacH8akY=wiFfYdH8Gipec8Eeeu0xXdbba9frFj0=OqFfea0dXdd9vqai=hGuQ8kuc9pgc9s8qqaq=dirpe0xb9q8qiLsFr0=vr0=vr0dc8meaabaqaciaacaGaaeqabaqabeGadaaakeaacqWGmbatdaWgaaWcbaGaeeOCaiNaeeyzauMaeeiDaqNaeeyDauNaeeOCaiNaeeOBa4gabeaakiabg2da9maalaaabaGaemitaW0aaSbaaSqaaiabigdaXaqabaGcdaqadaqaaiabdgeabnaaBaaaleaacyGGTbqBcqGGHbqycqGG4baEaeqaaOGaeiiFaWNaemOqai0aaSbaaSqaaiabbkhaYjabbwgaLjabbsha0jabbwha1jabbkhaYjabb6gaUbqabaaakiaawIcacaGLPaaaaeaacqWGTbqBdaWgaaWcbaGaeeOCaiNaeeyzauMaeeiDaqNaeeyDauNaeeOCaiNaeeOBa4gabeaakiabgEna0kGbcYgaSjabc6gaUjabikdaYaaaaaa@5BB9@

**Figure 2:**
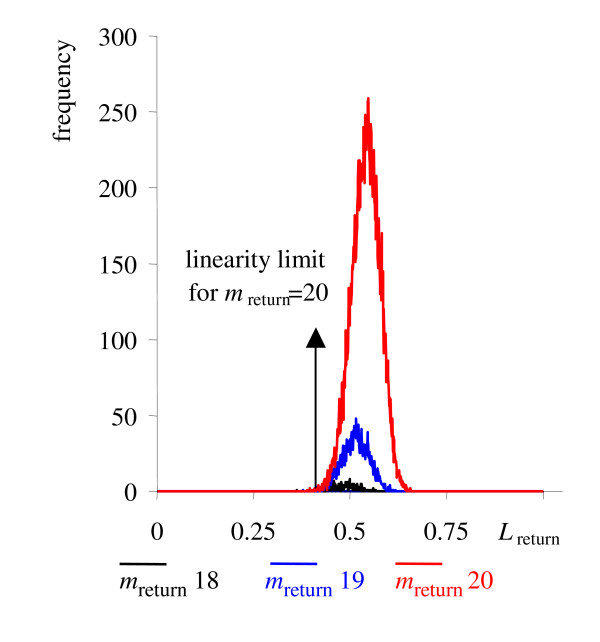
Determination of the linearity rejection region by Monte Carlo simulations. 3–150 samples were used from linear functions which were imposed by additional Gaussian noise. The example for *m *= 20 is shown, for which in some cases, fewer than 20 samples were returned due to outliers that were caused by the imposed technical error. For each of these *m*_return _values, adapted maximum likelihood limits were determined for which the null hypothesis, the existence of a linearity, would need to be rejected.

We now have two parameters, *m*_return _and *L*_return_, for which test statistics can be determined based on randomly selected true linear relationships. *A*_max _denotes the parameters for which the maximum likelihood is assumed. The distributions of *m*_return _and *L*_return _were assessed by Monte Carlo simulations: For each sample size ranging from 3 to 150 data points we have generated 25,000 random data sets, and test statistics were derived for each sample size *m*. The data set were generated by selecting an arbitrary linear function and a random selection of data points corresponding to this linear function. Technical errors were sampled from Gaussian distributions and added to the data points. After localizing the maximum value of *L*_1 _one determines all samples which belong to the corresponding linear function. Based on *L*_1 _and *m*_return_, the value of *L*_return _is determined as given above. The frequency distributions of *L*_return _for different values of *m*_return _are shown for the example of *m *= 20 samples (figure 2). Figure 2 demonstrates that the *L*_return _distributions varied for different *m*_return _values, and consequently, corresponding test statistics were established that set the limits for rejecting the null-hypotheses at a false negative error rate of ≤ 5% for each of the *m*_return _values.

### (5) Determination of false positive and false negative error rates

The degree of noise can be described in terms of the reliability that is defined as ratio of biological variance and total variance. The later is just the sum of biological and technical variance if both variances are not correlated. In that case the reliability of the measurement of metabolite *x *is given as

ρx=σbiol2(x)σbiol2(x)+σtech2(x)
 MathType@MTEF@5@5@+=feaafiart1ev1aaatCvAUfKttLearuWrP9MDH5MBPbIqV92AaeXatLxBI9gBaebbnrfifHhDYfgasaacH8akY=wiFfYdH8Gipec8Eeeu0xXdbba9frFj0=OqFfea0dXdd9vqai=hGuQ8kuc9pgc9s8qqaq=dirpe0xb9q8qiLsFr0=vr0=vr0dc8meaabaqaciaacaGaaeqabaqabeGadaaakeaaiiGacqWFbpGCdaWgaaWcbaGaemiEaGhabeaakiabg2da9maalaaabaGae83Wdm3aa0baaSqaaiabdkgaIjabdMgaPjabd+gaVjabdYgaSbqaaiabikdaYaaakiabcIcaOiabdIha4jabcMcaPaqaaiab=n8aZnaaDaaaleaacqWGIbGycqWGPbqAcqWGVbWBcqWGSbaBaeaacqaIYaGmaaGccqGGOaakcqWG4baEcqGGPaqkcqGHRaWkcqWFdpWCdaqhaaWcbaGaemiDaqNaemyzauMaem4yamMaemiAaGgabaGaeGOmaidaaOGaeiikaGIaemiEaGNaeiykaKcaaaaa@549C@

The average reliability can easily be obtained from the simulations, and hence, the degree of noise can well be described as

R=1−ρ¯x.
 MathType@MTEF@5@5@+=feaafiart1ev1aaatCvAUfKttLearuWrP9MDH5MBPbIqV92AaeXatLxBI9gBaebbnrfifHhDYfgasaacH8akY=wiFfYdH8Gipec8Eeeu0xXdbba9frFj0=OqFfea0dXdd9vqai=hGuQ8kuc9pgc9s8qqaq=dirpe0xb9q8qiLsFr0=vr0=vr0dc8meaabaqaciaacaGaaeqabaqabeGadaaakeaacqWGsbGucqGH9aqpcqaIXaqmcqGHsisliiGacuWFbpGCgaqeamaaBaaaleaacqWG4baEaeqaaOGaeiOla4caaa@352E@

We here assume that linear relationships between two metabolites are only confused by technical errors, but not by other biological factors, so the degree of noise here is only induced by technical errors. In order to test the algorithm described above, a data set was simulated that closely describes the problem. This model data set comprised 200 variables which were grouped into 20 clusters of equal size. All variables within a cluster were described by a linear relationship *y *= *ax *+ *b*, but between clusters, no linearity was modeled apart from random relationships. For each test, a different number of samples was taken to assume experimental data from metabolomic snapshots, with further and various levels of technical errors that were added to the modeled measurements. Technical errors were assumed to follow a Gaussian distribution. In total, the total data set was investigated for 19,900 pair-wise relationships of which 900 were modeled to be described by linear relationships in order to assess false positive and false negative error rates. The parameters were varied in an exhaustive permutation of the sample size from 3–150 samples and relative technical errors from 0.1–100%. Error rates were determined four times for each combination of ,number of samples' and ,technical errors', and the average of these four determinations was taken. Technical errors may be divided into the absolute error and a relative error. The absolute error, for example, is constituted by the resolution of an analytical instrument or constant background through chemical impurities of reagents and solvents. Such errors can be carefully controlled in validated chemical procedures and are usually less important than relative errors. In most cases in metabolomics, the total technical error is dominated by relative errors that relate to the true value, originating for example by sample storage, extraction and sample preparation procedures, and by cross-contamination and carry over between samples. Technical errors can be estimated by reproducing all sample preparation steps multiple times from small aliquots of a larger homogenized pool, and subsequent data acquisition. The magnitude of relative errors varies by the vulnerability of the compounds to be altered during the sample preparation and measurement process. However, for the sake of clarity, identical technical errors were used in the simulations for each pair of metabolites.

**Figure 3:**
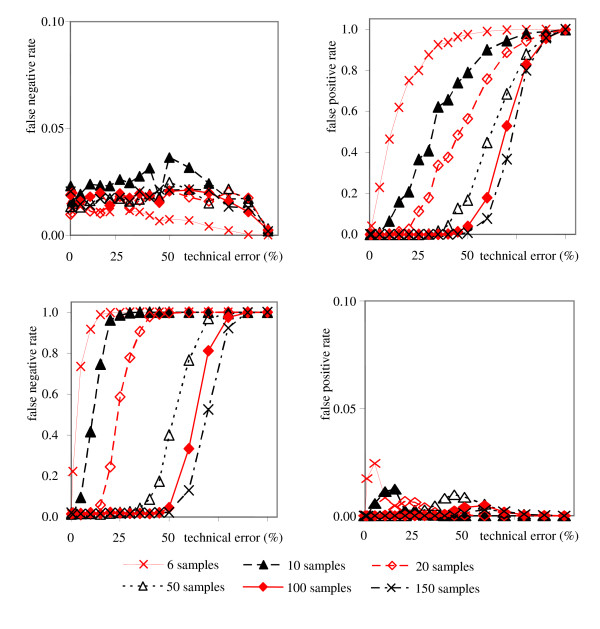
False negative and false positive error rates of the algorithm tested on simulated data in relation to the number of samples and the assumed technical errors, in % of the total variance. 900 pair-wise linear relationships between 200 metabolites were defined that were tested against the total of 19,900 potential linearities. Upper panel: Error rates without filter. Lower panel: error rates with filter.

The error rate of the algorithm is exemplified for selected sample numbers in figure 3 (upper panel), using the algorithm described so far. The null hypothesis used here was assuming the existence of a linear relationship between any pair of metabolites. This is in opposite to classical use of null-hypotheses, reversing the meaning of false positives and false negatives in our work. Therefore, in our case rejecting the null hypothesis when it is actually true means rejecting true linearities or generating false negative errors. It is important to note that the count for false negative detections (type I errors) stays below 5% except for sample numbers smaller than five. The minimal error rate corresponds to the limits that resulted from constructing the test statistics. For the false positive error rates (type II errors), a different trend is observed. Except for very low technical errors or large numbers of samples, the type II errors quickly exceed the 5% error thresholds. Generally, the sample size required to cope with higher technical errors rapidly increases for maintaining acceptable false positive rates. For high technical errors, the pattern between any two variables resembles a scatter around a constant value. In such cases, the number of false positive linearity detections increases because any constant value can be explained by a discretionary linear function. If higher limits were used for the parameters *L*_return _and *m*_return_, the 5% threshold for the false positive rates would be reached at higher technical error rates. However, simultaneously, the minimal error rate for of false negatives would increase. Consequently, type I and type II error rates could in principle be balanced by adapting the thresholds for *L*_return _and *m*_return _in a qualitative manner. Nevertheless, the total error rate can only be influenced by decreasing the technical error or increasing the number of samples taken into account.

**Figure 4:**
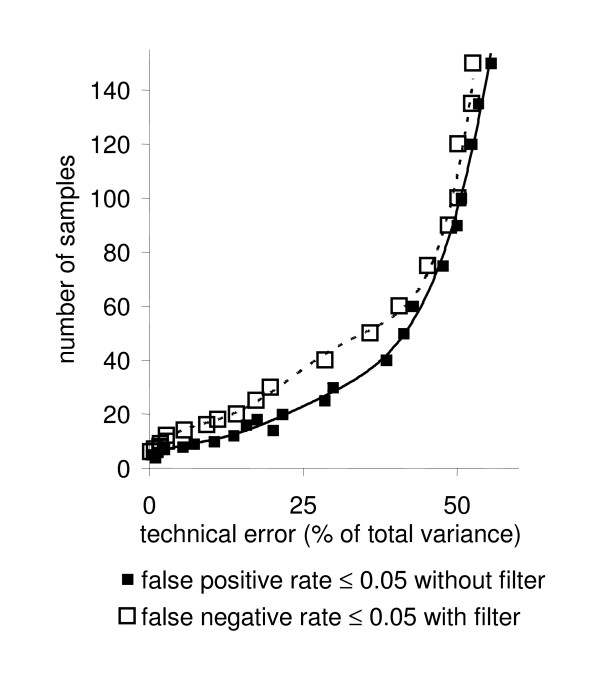
Number of samples required in relation to the assumed relative technical errors, if both false positive and false negative error rates are to remain below 0.05 (i.e. 5%). 900 pair-wise linear relationships between 200 metabolites were defined that were tested against the total of 19,900 potential linearities.

As outlined above, increasing levels of technical errors cause higher false positive error rates of detections of linearities. However, the number of false positive detections can be shifted towards false negative error rates, if desired for a specific biological study. Therefore, a filter has been developed that filters out all potential false positives (Additional File 4). The filter has been tested on the same simulated data as the algorithm before. Results are shown in figure 3 (lower panel) for false negative and false positive linearity detections. Compared to figure 3 (upper panel), a reverse order for false linearity discoveries was observed. Technical errors in metabolomics are usually in the range around 20–25%, although for certain compound classes, these may be lower. For a given biological situation, experimental biologists rarely use more than 10 biological replicates ('samples'), often even less. With other words, for such a combination of technical errors and low number of replicates, the algorithm yields either the accurate identification of all true linearities (without filter), but for the price of a high number of false positives, or the algorithm results in the full deletion of false positives (with filter), but for the price of not detecting a high number of the true linearities. We therefore have compared the results with and without filter in order to determine, how many samples would be needed to stay reliably at a false error rate ≤ 0.05 (i.e. 5%) for both false negatives and false positives. Figure 4 demonstrates that false positive detections without activating the filter obey a more favourable response to the combination of sample size and technical errors than the false negative error rates with active filter. Consequently, at technical errors of 20%, a minimum of about 20 samples is needed to stay reliably below 5% error rates for both false positive and false negative detections of linear metabolic relationships. This is an important result for practical use of this algorithm for robust generation of metabolomic networks. As default, the filter is not needed to be applied unless researches want to be very strict on the false positive error rates. The simulation presented here demonstrates that it is possible to remain at a total error rate of less than 5% if more than 20 samples are analyzed at the 20% technical error rate. The entirety of linear relationships of metabolites may subsequently be visualized as network graphs. Such graphs can be compared between different physiological or genetic conditions, in order to generate novel functional hypothesis on regulation of metabolic networks in a robust manner.

**Figure 5:**
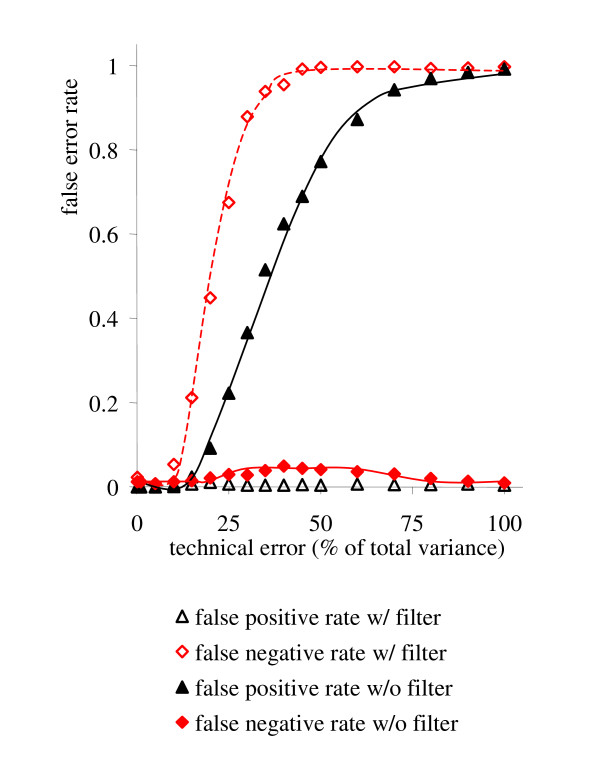
Influence of outliers on false negative and false positive error rates on a sample size of *m *= 20. In a similar manner to figure (3), simulated data were imposed by technical errors, but in addition, by the presence of outliers that were located in a 2–1000 *σ *distance from the linear function. The algorithm proved to be robust against such outliers.

The robustness of the algorithm was tested on a model dataset with 20 samples (figure 5). The thresholds for *L*_return _and *m*_return _were adjusted to tolerate an outlier rate of 5% (one of 20 samples). Outliers were modelled with a distance from 2 *σ *to 1000 *σ *away from the true linearity. It was found that outliers were generally easier recognized when these were very distant from the linearity. Despite the additional outliers, false positive and false negative error rates were found to almost identical as in figure 3. The number of false negative linearity detections remained below 5% without filter in all cases, and conversely, the false positive rate remained unchanged with activated filter. Hence, the use of Bayesian likelihood estimations enables robust detection and verification of linear relationships in an unbiased way and in complex datasets. If more outliers are present in a data set, these may actually constitute several local likelihood maxima as shown in figure 1C (lower panel) or in the figures in Additional File 2. Such additional linear relationships may be revealed if several outliers follow a different linear function and hence yield unexpected hypotheses of cellular regulation. Finding and validating such multiple linearities has so far been hard to accomplish with classical tools but is now amenable with the algorithm presented here. The algorithm has been implemented in a stand alone software solution. For data sets of a size of 200 variables × 150 samples, robust linearity networks are generated in around 10 min computing time using a 512 MB RAM and 3.5 GHz personal computer. The actual computing time will vary from 3.5–15 min, depending on the actual linearity structure of the data set. Improved implementations of the algorithm, specifically for the search of global likelihood maxima, may certainly be worked out more effectively with respect to computational run time. However, acquiring metabolomic data of the size of 150 samples (from growth of biological organisms, harvesting, sample processing, data acquisition to data processing) will take time on the order of weeks which surely justifies computational efforts on standard personal computers on the order of minutes.

## Conclusion

Use of the technical error concomitant with a maximum likelihood assessment of linearity parameters and verification by simulated test statistics enables a robust detection and verification of liner relationships in complex data sets. An implementation of this algorithm will enable biologists to calculate and compare linearity networks in metabolomic or other multivariate data sets, from which biological hypotheses may be derived. The algorithm can be modified with respect to the ratio of type I and type II errors depending on the biological focus of a study. It is highly advised to use more than 20 biological replicates for each condition that is to be tested in a biological experimental design of *genotypes x environments *(*G x E*), unless advances in analytical chemistry and instrumentation decrease the overall technical error to very low levels, i.e. below 5%. Even the existence of more than one linear relationship per pair of variables can be detected using the maximum likelihood algorithm, which has so far been hard to compute with classical approaches.

## Authors' contributions

FK has worked out, tested and implemented the algorithm. MH had initially advised on the mathematics of likelihood estimations. JB eventually revised and improved the mathematical description of the algorithm and contributed to writing the paper. OF conceived the study, participated in developing and testing the algorithm and drafted and wrote the manuscript.

## Supplementary Material

Additional File 1

Additional File 2

Additional File 3

Additional file 4
