# Supplementary material for: Robust detection and verification of linear relationships to generate metabolic networks using estimates of technical errors
Source: BMC Bioinformatics. 2007 May 21;8:162. doi: 10.1186/1471-2105-8-162 (PMC1894643; doi:10.1186/1471-2105-8-162)
Supplement: Additional File 1 [file 1471-2105-8-162-S1.pdf]

(Supplement 1) Proof of the theorem

We will outline the proof of the theorem for the case of n metabolite measurements $x = (x_1, ..., x_n)$ and a single hyperplane defined by the equation

(S1) $$\alpha_1 x_1 + ... + \alpha_n x_n = \beta .$$

However, the proof for a general surface defined by N equations exactly goes along the lines of the hyperplane case. It essentially leans on the orthogonality condition that leads to a decoupling of the terms that come from the different hyperplane equations (S1). The likelihood of the n metabolite concentrations to lie on a hyperplane is given by the convolution of the corresponding error distribution with the density that describes the surface,

(S2) $$p(x\,|\,\rho) = N\int_{R^n} \exp\left(-\frac{1}{2}(x-x')^t \Sigma^{-1}(x-x') \right)\rho(x')\, d^n x' .$$

Here ρ is the density describing the hyperplane and N a suitable normalization constant. We will take advantage of the trick to smear out the delta-distribution-like density to a family of Gauss functions,

(S3) $$\rho(x') = \delta(\alpha^t x' - \beta) = N' \lim_{\varepsilon\to 0} \frac{1}{\varepsilon} \exp\left(-\frac{(\alpha^t x' - \beta)^2}{2\varepsilon^2} \right).$$

This leads to significant simplifications: The integration can be extended over the whole n-dimensional space and the projection on the hyperplane can be done after integration, namely by letting the variance of the Gauss functions approach zero. In what follows we do not need to care about several factors that appear during the manipulations. We absorb them into the normalization constant which can be restored by the condition

(S4) $$p(x\,|\,\rho) = 1 \quad \text{for } \alpha^t x = \beta$$

at the end of the calculations. Let S be a square root of the positive definite, symmetric matrix Σ. As first step towards the calculation of the integral (S5) we perform a change of variables to $y = S^{-1}(x' - x)$ resulting in

(S5) $$p(x\,|\,\rho) = N\int_{R^n} \exp\left(-\frac{1}{2} y^t y \right)\rho(x + Sy)\, d^n y .$$

Next, we put in the representation for the density and obtain

(S6) $$p(x\,|\,\alpha,\beta) = N \lim_{\varepsilon\to 0} \int_{R^n} \exp\left(-\frac{1}{2} y^t A y + b^t y + c \right)$$

wherein

(S7) $$A = I + \varepsilon^{-2}\alpha S^2 \alpha^t , \quad b = \varepsilon^{-2}(\alpha^t x - \beta) S\alpha , \quad c = -\frac{1}{2}\varepsilon^{-2}(\alpha^t x - \beta)^2 .$$

Execution of the Gaussian integration yields the result

(S8) $$p(x\,|\,\alpha,\beta) = N \lim_{\varepsilon\to 0} \frac{1}{\varepsilon\sqrt{\det A}} \exp\left(b^t A^{-1} b + c \right).$$

To evaluate expression (S8), we consider the vector $a := S\alpha$ and the matrix aa^t that projects on a straight line in direction of a. We obtain $A = I + \varepsilon^2 aa^t$, $\det A = 1 + \varepsilon^{-2} a^t a$, the eigenvector relation $Aa = (1 + \varepsilon^{-2} a^t a)a$, and $a^t A^{-1} a = a^t a\,(1 + \varepsilon^{-2} a^t a)^{-1}$. With these results in hand we are in position to evaluate the exponent of (S8),

(S9) $$\begin{aligned} b^t A^{-1} b + c &= \frac{1}{2}\varepsilon^{-4}(\alpha^t x - \beta)^2 \frac{a^t a}{\varepsilon^{-2} a^t a + 1} - \frac{1}{2}\varepsilon^{-2}(\alpha^t x - \beta)^2 \\ &= \frac{1}{2}\varepsilon^{-2}(\alpha^t x - \beta)^2 \left(\frac{a^t a}{a^t a + \varepsilon^2} - 1 \right) \\ &= -\frac{1}{2}\frac{(\alpha^t x - \beta)^2}{a^t a + \varepsilon^2} \end{aligned}$$

Finally, we reinstate the expression for the exponent in (S8) and execute the limit. By taking into account the normalization condition we obtain the final result

(S10) $$p(x\,|\,\alpha,\beta) = \exp\left(-\frac{1}{2}\frac{(\alpha^t x - \beta)^2}{\alpha^t \Sigma \alpha} \right).$$
