# Supplementary material for: Robust detection and verification of linear relationships to generate metabolic networks using estimates of technical errors
Source: BMC Bioinformatics. 2007 May 21;8:162. doi: 10.1186/1471-2105-8-162 (PMC1894643; doi:10.1186/1471-2105-8-162)
Supplement: Additional File 2 [file 1471-2105-8-162-S2.pdf]

(Supplement 2) Impact of the constant *c* on the optimization problem

We consider the measurements of two metabolites

(S11) $$x_j = x'_j + e_{x,j}$$

(S12) $$y_j = y'_j + e_{y,j}$$

with uncorrelated technical errors modelled by the distributions

(S13) $$\rho_x(e_{x,j}) = N \exp\left(-\frac{1}{2}\frac{e_{x,j}{}^2}{\sigma_x{}^2}\right), \quad \rho_y(e_{y,j}) = N \exp\left(-\frac{1}{2}\frac{e_{y,j}{}^2}{\sigma_y{}^2}\right).$$

Then, the adapted likelihood function for a linear relationship

(S14) $$y' = ax' + b$$

is given by

(S15) $$L_c\left(a,b \mid \{x_j, y_j\}\right) = \sum_{j=1}^{m} \ln\left(l_j(a,b \mid x_j, y_j) + c\right)$$

whereto each of the samples contributes with

(S16) $$l_j\left(a,b \mid x_j, y_j\right) = \exp\left(-\frac{1}{2}\frac{(y_j - ax_j - b)^2}{a^2\sigma_x{}^2 + \sigma_y{}^2}\right).$$

In what follows we analyze a simulated data set of 40 measurements that is shown in figure S2.1. In this example data, the relation between the concentrations of the metabolites depends on the sample and is described by one of two linear models Hyp1 and Hyp2.

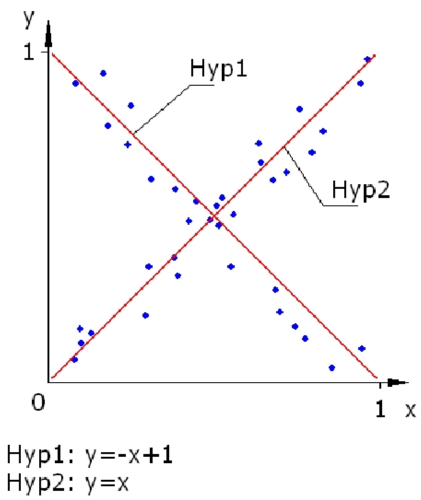

Figure S2.1: Simulated measurements of the metabolites *x* and *y* that can be described either by the linear model Hyp1 or Hyp2, each represented by 20 measurements that were confused by random technical errors.

We study the following four cases for the impact of the constant *c* using two hypothetical relationships Hyp1 and Hyp2 of the metabolites concentrations *x* and *y*:

Case 1: $c = 0$

Case 2: $0 < c < 1$

Case 3: $c = 1$

Case 4: $c > 1$

Case 1

For c = 0 any technical errors of a measurement leads to a decrease of the likelihood function. L is always negative except for l_j=1. There is exactly one global maximum which is reached when this decrease is minimal. A heatmap of the likelihood function in dependence of slope and intercept is shown in figure S2.2. The most likely hypothesis is very similar to the result of a normal regression: it is a straight line with slope 0 and intercept 0.5 and there is no evidence for the existence of two different linear relationships. Conversely, without using the constant c, data have to be assumed to refer only to one linearity, and hence would need to be grouped or clustered according to assumed biological factors.

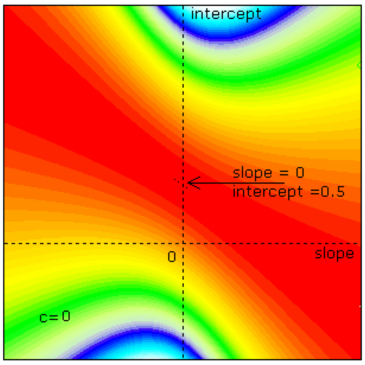

Figure S2.2: Heatmap of likelihood function in dependence of slope and intercept of the linear model. The likelihood increases from white to cyan, blue, green, yellow, orange to red. The global maximum is marked by a black dot, further indicated by an arrow.

Case 2

For 0 < c < 1, three values of c were simulated with $c = 10^{-13}$, $c = 10^{-7}$ and $c = 10^{-3}$. With increasing c the heatmap of L progresses to represent two maxima and now enables unbiased data exploration by detection of more than one linear relationship, see figure S2.3.

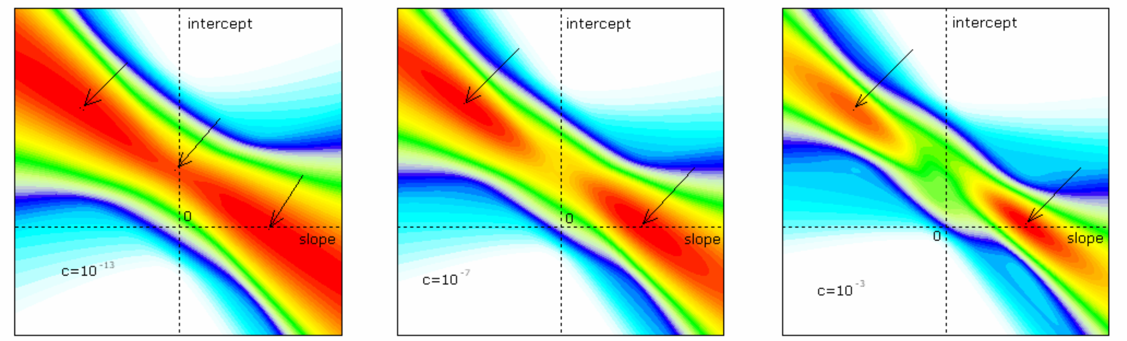

Figure S2.3: Progression of the heatmap of L_{norm} by increasing the constant c. Left panel: $c = 10^{-13}$, mid panel $c = 10^{-7}$, right panel $c = 10^{-3}$.

Case 3

For c = 1 the minimum value of the likelihood function is L = 0. A measurement that is exactly in accordance with the investigated linear relationship contributes to it with $l_j + c = 2$. Therefore, the maximal contribution to L is max(L) = m ln(2). Consequently, each measurement increases L by a contribution between 0 and ln(2), distinct from cases (1) and (2) where outliers led to an decrement of the likelihood function. This is also the basic reason why the algorithm is not only robust against outliers but also can detect separate linear relationships in a single complex data set. The two detected likelihood relationships Hyp1 and Hyp2, see figure S2.4.

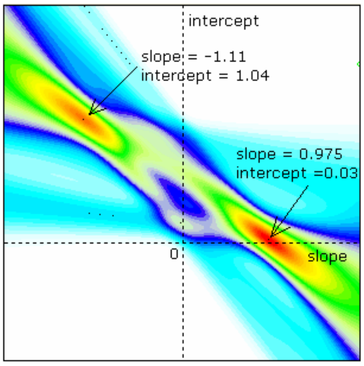

Figure S2.4: Heatmap of the likelihood function for c = 1. Two maxima are detected which reflect the modeled linear hypotheses Hyp1 and Hyp2.

Case 4

Switching from c = 1 to c > 1, no drastic changes are observed in the heatmap of the likelihood function. The results for $c = 10^3$, $c = 10^7$ and $c = 10^{13}$ are shown in figure S2.5.

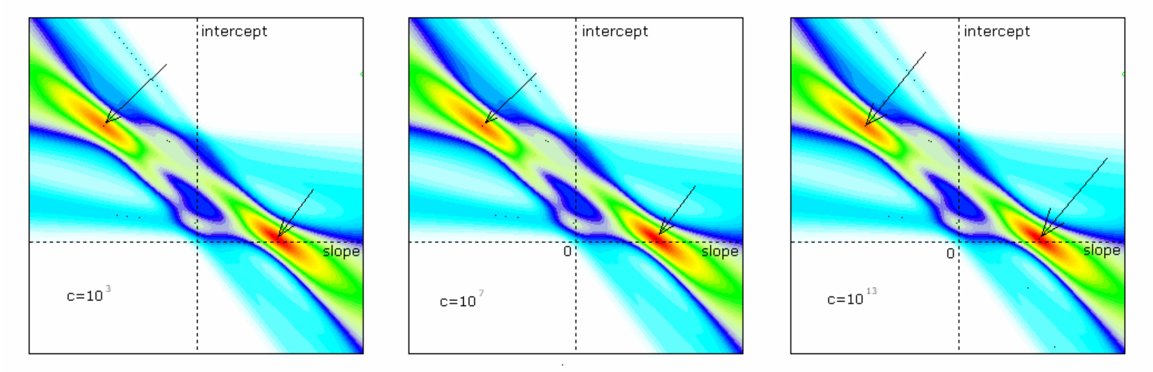

Figure S2.5: Exemplification of the distribution of L_{norm} when altering the constant *c to values c>1*. Left panel: $c = 10^3$, mid panel $c = 10^7$, right panel $c = 10^{13}$. The topology of likelihood distributions and the localization of intercept and slope parameters remains essentially identical to c = 1.
