# Supplementary material for: Robust detection and verification of linear relationships to generate metabolic networks using estimates of technical errors
Source: BMC Bioinformatics. 2007 May 21;8:162. doi: 10.1186/1471-2105-8-162 (PMC1894643; doi:10.1186/1471-2105-8-162)
Supplement: Additional File 3 [file 1471-2105-8-162-S3.pdf]

(Supplement 3) The algorithm to solve the optimization problem

The search for the most likely hypothesis (maximum of *L*) between two variables *(x, y)* is divided into the subsequent two steps because the hypotheses space may be comprised of several maxima.

1. Estimation of the starting value of linearity parameters
2. Approximation to the global maximum

The estimation of the search starting value is performed using a modified regression analysis with using weighted data according to the assumptions of the technical errors. Regression analysis is known to lead to different results depending on which variable of a variable pair is regarded as independent. Therefore, our algorithm applies regression to both variables in a consecutive manner. Unfortunately, regression analysis is also known to be sensitive against outliers who may lead to a starting value far from the overall maximum. In order to minimize the effects of this problem, the data set is analyzed in seven modifications, each of which excludes certain pairs of data (table 1).

Table 1: Modifications of original input data sets to be used for regression analysis.

modification 1	original input data set
modification 2	input data set excluding the *M* highest *x*-values
modification 3	input data set excluding the *M* smallest *x*-values
modification 4	input data set excluding the *M* highest and *M* smallest *x*-values
modification 5	input data set excluding the *M* highest *y*-values
modification 6	input data set excluding the *M* smallest *y*-values
modification 7	input data set excluding the *M* highest and *M* smallest *y*-values

There are 14 different results based on the two variables and seven modifications. The assumption using these modifications is that outliers get potent if these are extreme. The final estimate for the starting parameter value for linearity maximum likelihood is determined by determining the highest value of *L*.

For generating modified data sets, *M* was arbitrarily selected at 10% of the rounded total number of variable pairs, but at least 1 if for numbers of data pairs >4. In case more than 10% of the variable pairs show extreme deviation in regression analysis, it can be assumed that there is additional biological information.

After selecting the starting value, the global linearity maximum is approximated (see figure S4.1).

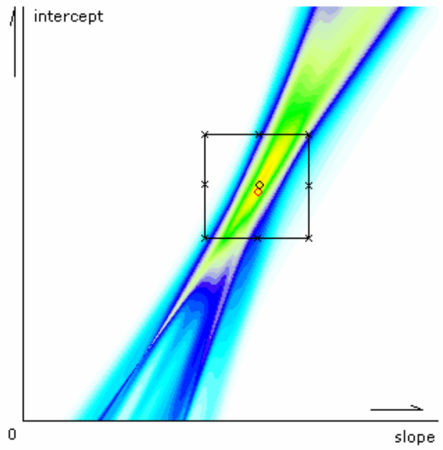

Figure S3.1: Cartoon displaying the algorithm for searching the global maximum of the likelihood distribution of a given linearity.

The starting value is given as black circle with 8 points that mark the initial scan points for searching any *L* that has a higher likelihood than the starting value. The vector between starting value and initial scan point defines

the search direction, with a random selection of one of the 8 scan points to start the approximation. The likelihood search continues with the next scan point and counts the events if smaller linearity likelihoods result. If likelihoods increase at any scan point, this scan point is chosen as a starting point for a new search with a square of 8 new scan points. The search direction is maintained and the procedure is repeated. In case the number of smaller likelihood events sum to 8 for a given search square it must be assumed that the global maximum is within the square. The search interval is divided by a factor of 2 and the approximation process is repeated. The search square size for start and abortion of the approximation can be uniform because all data have been normalized. Experiments with this approximation have resulted with a starting square edge size of 1 and an abortion edge size of 0.0005.
