# Supplementary material for: Robust detection and verification of linear relationships to generate metabolic networks using estimates of technical errors
Source: BMC Bioinformatics. 2007 May 21;8:162. doi: 10.1186/1471-2105-8-162 (PMC1894643; doi:10.1186/1471-2105-8-162)
Supplement: Additional file 4 [file 1471-2105-8-162-S4.pdf]

(Supplement 4) Definition of the filter

False positive detections of hypothetical linearities are increasing at increasing technical errors but may be shifted towards false negative error rates, if needed. Such a case might be wished if false positive detections would hamper biological interpretations in a more severe way than if real linearities would stay undetected. Therefore, a filter has been developed using a calculation of a characteristic value *t* to suppress false positive errors (S17).

(S17) $$t = \frac{\sqrt{\left|\sum_{j=1}^{m}(\mu_x - x_j)(\mu_y - y_j)\right|}}{\sqrt{\sum_{j=1}^{m}\sigma_{tech}(x_i)\sigma_{tech}(x_j)}}$$

μ_x mean of all measurements of metabolite *x*

μ_y mean of all measurements of metabolite *y*

$\sigma(x_j)$ standard deviation of the technical error distribution of metabolite *x* when measured in sample *j*

$\sigma(y_j)$ standard deviation of the technical error distribution of metabolite *y* when measured in sample *j*

The numerator in formula (1) comprises a modified form of the covariance. For the case of an exclusive technical error variance and an infinite sample number, *t* approximates zero because then the covariance will converge to zero. The denominator estimates the maximal covariance which can result from the assumed technical error. An upper limit for *t* can be determined by simulating a technical error distribution around a constant value. If the actual *t* is larger than the theoretical threshold for *t*, the hypothesis can be rejected that the corresponding covariance may have originated at random through the technical error. For $t < t_{limi}$, false positive discovery rates cannot be eliminated. In figure 1, simulation results for parameter *t* are given for a variation of sample numbers. The simulation set consists of the following steps:

- All metabolite concentrations are set to zero
- Each metabolite concentration is superimposed by technical error sampled from a Gaussian distribution
- *t* is determined according to formula (1)

The simulation has been repeated at least 55.000 times.

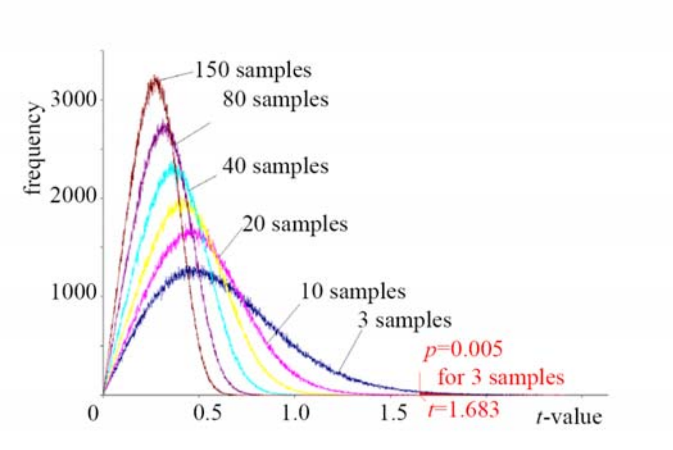

Figure S4.1: Simulation results for parameter according to equation (1), varying the number of samples in a data set. For each number of samples, one million simulations were carried out. As example, the threshold for t a p=0.005 and three samples is given.

The value of *t* has been adjusted in a way that the detection rate of false positives remains below 5% independently from the degree of noise.
